# Soil *nifH*-harboring community assemblage varies across pecan cultivars

**DOI:** 10.3389/fmicb.2025.1716240

**Published:** 2026-01-07

**Authors:** Junping Liu, Hankun Wang, Yujie Tang, Jiashu Bao, Pengpeng Tan, Fangren Peng

**Affiliations:** 1 Institute of Jiangxi Oil-tea Camellia and College of Pharmacy and Life Science, Jiujiang University, Jiujiang, Jiangxi Province, China; 2 Co-Innovation Center for Sustainable Forestry in Southern China, Nanjing Forestry University, College of Forestry and Grassland, Nanjing, Jiangsu Province, China

**Keywords:** pecan cultivars, nitrogenase activity, *nifH*-harboring community, rhizosphere microbiome, microbial network

## Abstract

**Introduction:**

This study focused on three pecan (*Carya illinoinensis*) cultivars (‘Pawnee’, ‘Mahan’, and ‘Jinhua’), systematically assessing variations in soil nitrogenase activity, characteristics traits of *nifH*-harboring microbial communities across these cultivars.

**Methods:**

Using high-throughput sequencing technology, differences in the diversity, community composition, and network structure of *nifH*-harboring communities in the rhizosphere and bulk soils of pecan were examined across cultivars.

**Results:**

Both cultivar type and soil compartment had significant effects on nitrogenase activity (*p* < 0.01). Among the three cultivars, ‘Mahan’ exhibited the highest soil nitrogenase activity in both its rhizosphere and bulk soils relative to the other two cultivars. Notably, rhizosphere soils across all cultivars displayed significantly stronger soil nitrogenase activity than their bulk soil counterparts. ‘Mahan’ harbored significantly higher microbial *α*-diversity (Sobs, Shannon, and Chao indices) than other cultivars (*p* < 0.05). *β*-diversity analysis revealed marked community divergence among cultivars, with the most pronounced differences observed in bulk soils. Specifically, the bulk soil of ‘Jinhua’ harbored a distinct microbial signature, marked by significant enrichment of *Cyanobacteria* and depletion of *Alphaproteobacteria*. Linear discriminant analysis effect size (LEfSe) further identified *Rhizobiales* and *Burkholderiales* as distinct biomarkers for the rhizosphere and bulk soils of ‘Jinhua’, respectively (*LDA* score > 4.0, *p* < 0.05). Microbial co-occurrence network analysis showed that the bulk soil of ‘Jinhua’ harbored the most complex microbial interaction network, characterized by the highest number of edges, and average connectivity. In co-occurrence networks, *Azohydromonas*, *Bradyrhizobium*, *Azoarcus*, *Rhodomicrobium* were found as the keystone taxa in maintaining network stability.

**Discussion:**

This research elucidates the regulatory roles of pecan cultivars in shaping soil nitrogen fixation functions and microbial community assembly, providing valuable practical implications for precision microbiome management in pecan production.

## 1 Introduction

Nitrogen is a critical macronutrient essential for plant growth and development. However, its bioavailability in agricultural systems is frequently constrained by natural biogeochemical processes (Guo et al., 2023). Biological nitrogen fixation (BNF), mediated by specialized diazotrophic microorganisms, converts atmospheric dinitrogen (N₂) into biologically available ammonia. This process enhances soil fertility and reduces dependence on synthetic fertilizers in agricultural systems (Guo et al., 2023). The *nifH* gene, which encodes the iron protein subunit of nitrogenase, has been widely used as a reliable phylogenetic marker for investigating the diversity and distribution of diazotrophic communities in terrestrial ecosystems (Gaby John et al., 2018). Characterizing the diversity and abundance of soil *nifH*-harboring communities is essential for understanding the mechanisms of nitrogen cycling in ecosystems.

Pecan (*Carya illinoinensis*), a high-value nut tree cultivated worldwide in temperate regions, exhibits strong nitrogen-dependent growth responses that directly influence its yield potential (Wells, 2021; Pokhrel et al., 2025). While previous studies have extensively examined the agronomic effects of fertilization regimes on pecan productivity (Tong et al., 2024; Pokhrel et al., 2025), the functional role of indigenous diazotrophic communities associated with different cultivars remains poorly understood. It is well-documented that plant genotype can shape rhizosphere microbiome assembly through mechanisms such as root exudation (Rheault et al., 2020; Yang et al., 2022). For instance, studies in crops like sugarcane and cotton have demonstrated cultivar-specific differences in diazotrophic communities (Dong et al., 2018; Yang et al., 2022). Therefore, we hypothesize that distinct pecan cultivars may selectively enrich specific *nifH*-harboring microbial taxa, potentially influencing nitrogen fixation capacity.

Current knowledge of pecan-associated soil microbiomes has primarily focused on general bacterial and fungal communities (Ren et al., 2023; Ren et al., 2024), leaving a critical knowledge gap regarding diazotrophic assemblages. Specifically, the mechanisms underlying cultivar-specific recruitment of diazotrophic communities and their functional implications in pecan orchards have not been systematically investigated. Addressing this gap could provide insights into cultivar-specific microbial interactions that enhance nitrogen availability and reduce fertilizer dependency.

Specifically, we aimed to (1) characterize the diversity and composition of soil *nifH*-harboring microbial communities associated with different pecan cultivars and (2) quantitatively assess the soil nitrogen fixation potential of different pecan cultivars. We hypothesized that (i) distinct pecan cultivars harbor significantlybdifferent *nifH*-harboring microbial communities, and (ii) specific keystone microbial taxa drive cultivar-dependent variations in soil nitrogen fixation through deterministic community assembly processes. The findings are expected to provide a theoretical basis for developing microbiome-based strategies for sustainable pecan production, offering new insights for precision orchard management.

## 2 Materials and methods

### 2.1 Study site and soil sampling

This study was conducted in the Pecan Experimental Base of Nanjing Forestry University, Jurong city (J), Jiangsu Province, one of the main pecan cultivation regions in China with a subtropical humid monsoon climate and neutral-acid luvisol according to the soil taxonomy of the FAO. All experimental trees were consistent in key growth conditions: tree age (7 years), planting spacing (6 m × 8 m), management practices (uniform pruning, systematic weed control, and integrated pest control), and fertilization regime (annual application of organic compost). Rhizosphere (RS) and bulk soil (BS) samples were collected from three pecan cultivars, ‘Pawnee’ (P), ‘Mahan’ (M), and ‘Jinhua’ (J). Accordingly, six experimental groups were established: JPBS, JPRS, JMBS, JMRS, JJBS, and JJRS.

Six small plots (20 × 20 m^2^) were selected, with an inter-plot spacing of ≥ 50 m to avoid mutual interference. Each plot served as an independent biological replicate (n = 6). Within each plot, three uniformly distributed trees were chosen for soil mixture sampling (minimum spacing of 12 m between trees). From each tree, soil samples were collected from four directions (east, south, west, and north) and homogenized. Ultimately, each composite soil sample was composed of soil from 12 sampling points. Six composite soil samples were collected per group, with a total of 36 samples analyzed across all groups (total *n* = 36). Within a 0.5 m radius from the tree trunk, the surface humus was removed, and soil cores containing roots were collected using a soil auger. As pecan is a deep-rooted species, soil sampling was conducted at increased depths (0–60 cm) to ensure rhizosphere coverage. Following the identification of the pecan root system, rhizosphere soil was operationally defined as the soil firmly adhering to the root surface after gentle shaking to remove loose aggregates. This collected layer corresponds to soil within approximately 1–3 mm of the root surface. Concurrently, bulk soil was collected from areas located at least 10 cm away from any fine roots (0–60 cm depth). To avoid rhizosphere influence, the absence of roots was verified by visual inspection prior to sampling. All collected samples were immediately sealed in sterile polyethylene bags, clearly labeled, and stored in dry ice-cooled insulated containers to maintain biological integrity during transport to the laboratory. Upon arrival, each soil sample was sieved (2 mm mesh) and subdivided under aseptic conditions. One aliquot for DNA extraction and molecular analysis was stored at −80 °C. The other aliquot for soil physicochemical characterization was stored at 4 °C. prior to sampling, all tools were sterilized to eliminate potential microbial contamination.

### 2.2 Determination of soil nitrogenase activity

Soil biological nitrogen fixation (BNF) activity was assessed by measuring nitrogenase activity using the acetylene reduction assay (ARA), following a modified protocol based on the approach reported from published research (Qi et al., 2022). Briefly, 10 g of fresh soil (equivalent to 8.1 ± 0.06 g dry soil) was placed in a 20 mL penicillin bottle. All samples were maintained at their natural moisture content during the pre-incubation phase without additional water supplementation, but comparability within the experimental group was ensured by subjecting all samples to identical pre-treatment procedures. Samples were pre-incubated at 30 °C in the dark for 24 h with perforated breathable film to allow microbial acclimation. Following pre-incubation, the parafilm was replaced with a butyl rubber stopper and an aluminum-plastic cap to ensure airtight conditions. Using a syringe, 1 mL of gas was extracted from the bottle, and 1 mL of acetylene (C_2_H_2_) was injected (headspace volume: 10 mL). All bottles were gently shaken to ensure uniform gas distribution before incubation. Blank vials without soil substrate were included as negative controls in each batch to account for any ethylene background. Samples were then incubated at 30 °C in the dark under static conditions for 48 h. After incubation, 1 mL of headspace gas was sampled to measure the relative concentration of ethylene (C_2_H_4_).

Gas chromatography (GC) analysis was performed under the following optimized conditions: flame ionization detector (FID) temperature = 220 °C; capillary column (HP-PLOT Q, 30 m × 0.32 mm × 20 μm) temperature = 80 °C (isothermal); carrier gas = high-purity nitrogen (N₂, 99.999%) with a flow rate of 1.0 mL/min; fuel gas = high-purity hydrogen (H₂, 99.999%); auxiliary gas = compressed air. GC calibration was conducted using a series of C₂H₄ standard solutions (0.01–10 nmol/mL) to establish a standard curve (R^2^ ≥ 0.999), with a detection limit of 0.01 nmol. Each sample was injected in triplicate to ensure analytical precision. Nitrogenase activity was expressed as the amount of C_2_H_4_ produced per gram of fresh soil per hour (nmol C₂H₄ g^−1^ fresh soil h^−1^).

### 2.3 High-throughput sequencing of *nifH*-harboring community

Total DNA was extracted from 0.5 g of soil using the FastDNA SPIN Kit for Soil (MP Biomedicals, CA, United States) following the manufacturer’s protocol. Extraction blanks (negative controls) were included in parallel to monitor potential contamination during the DNA extraction process. DNA integrity was assessed by 1% agarose gel electrophoresis, while purity and concentration were determined using a NanoDrop 2000 spectrophotometer (Thermo Scientific, Wilmington, United States). The *nifH* gene of nitrogen-fixing microorganisms was amplified using the primers: nifHF: 5’-AAAGGYGGWATCGGYAARTCCACCAC-3′, nifHR: 5’-TTGTTSGCSGCRTACATSGCCATCAT-3′. PCR conditions and amplification procedures followed previously described methods (Fan et al., 2019). Each sample was amplified in triplicate, and PCR products from the same sample were pooled. PCR negative controls (with no template) were also performed to ensure the absence of contamination during amplification. The target fragments were excised from a 2% agarose gel and purified using the AxyPrep DNA Gel Extraction Kit (Axygen Biosciences, Union City, CA, United States). Purified amplicons were sequenced on an Illumina MiSeq PE300 platform (Illumina, San Diego, United States) at Majorbio Bio-Pharm Technology Co., Ltd. (Shanghai, China). The raw sequencing data have been deposited in the National Center for Biotechnology Information (NCBI), Sequence Read Archive (SRA) under accession number PRJNA907477.

Raw paired-end reads were first demultiplexed, quality-filtered using fastp (v0.20.0), and merged into contiguous sequences with FLASH (v1.2.7). Subsequently, operational taxonomic units (OTUs) were clustered at a 97% sequence similarity threshold using UPARSE (v7.1), with chimeric sequences identified and removed during this process. OTUs that aligned to chloroplast or mitochondrial sequences were further excluded to eliminate non-target contaminants. Following this, all samples were rarefied to the minimum number of sequences observed across the dataset to standardize sequencing depth. The cleaned sequence data were then aligned against the *nifH* functional gene database (Version 7.3)^1^ using a 70% alignment threshold for taxonomic and functional annotation. This confidence threshold was selected as it is widely adopted in *nifH* gene studies to balance classification precision with the retention of sufficient sequences for ecological analysis, given the comparatively smaller and less comprehensive nature of the *nifH* gene reference database. Prior to downstream statistical analysis, the sequencing data were subjected to minimum sequence normalization to ensure consistency across samples.

### 2.4 Statistical analysis

After rarefaction, the coverage index of all samples reached above 95% (Supplementary Figure 1), indicating that the sequencing depth was sufficient to capture the majority of the diazotrophic microbial composition and could be used for subsequent diversity analyses. Because the data did not meet the assumptions for parametric tests, the Scheirer–Ray–Hare test was used to evaluate the effects of cultivar., niche, and their interaction on *α*-diversity. Post–hoc pairwise comparisons were performed using Kruskal-Wallis test, with Bonferroni correction applied to control the false-discovery rate in multiple testing. NMDS was carried out with 999 random starts using the vegan package (v2.6–6) to ensure the stability of the ordination. A two-factor PERMANOVA (adonis2 function, 999 permutations) was conducted to test the overall effects of cultivar., niche, and their interaction on community structure. Homogeneity of multivariate dispersions was tested with the betadisper function.

To evaluate differences in soil nitrogenase activity, analysis of two-way variance (ANOVA) and paired t-tests were applied, with nitrogenase activity further visualized using GraphPad Prism 8. For microbial co-occurrence analysis, Spearman’s correlation was calculated between OTUs, and all correlations were adjusted for multiple testing with the False Discovery Rate (FDR) correction to control false positives. Statistically robust correlations were used to construct co-occurrence networks, which were subsequently analyzed with the igraph package (v0.9.2). It should be noted that these networks reflect inferred associations based on correlation statistics; while they suggest potential ecological linkages, they do not constitute direct evidence of biological interaction or causality. Additional bioinformatics steps, including sequence quality control, OTU clustering, taxonomic annotation, compositional analysis, and visualization were performed on the Majorbio Cloud Platform.^2^

## 3 Results

### 3.1 Soil nitrogenase activity of different pecan cultivars

**Figure 1:**
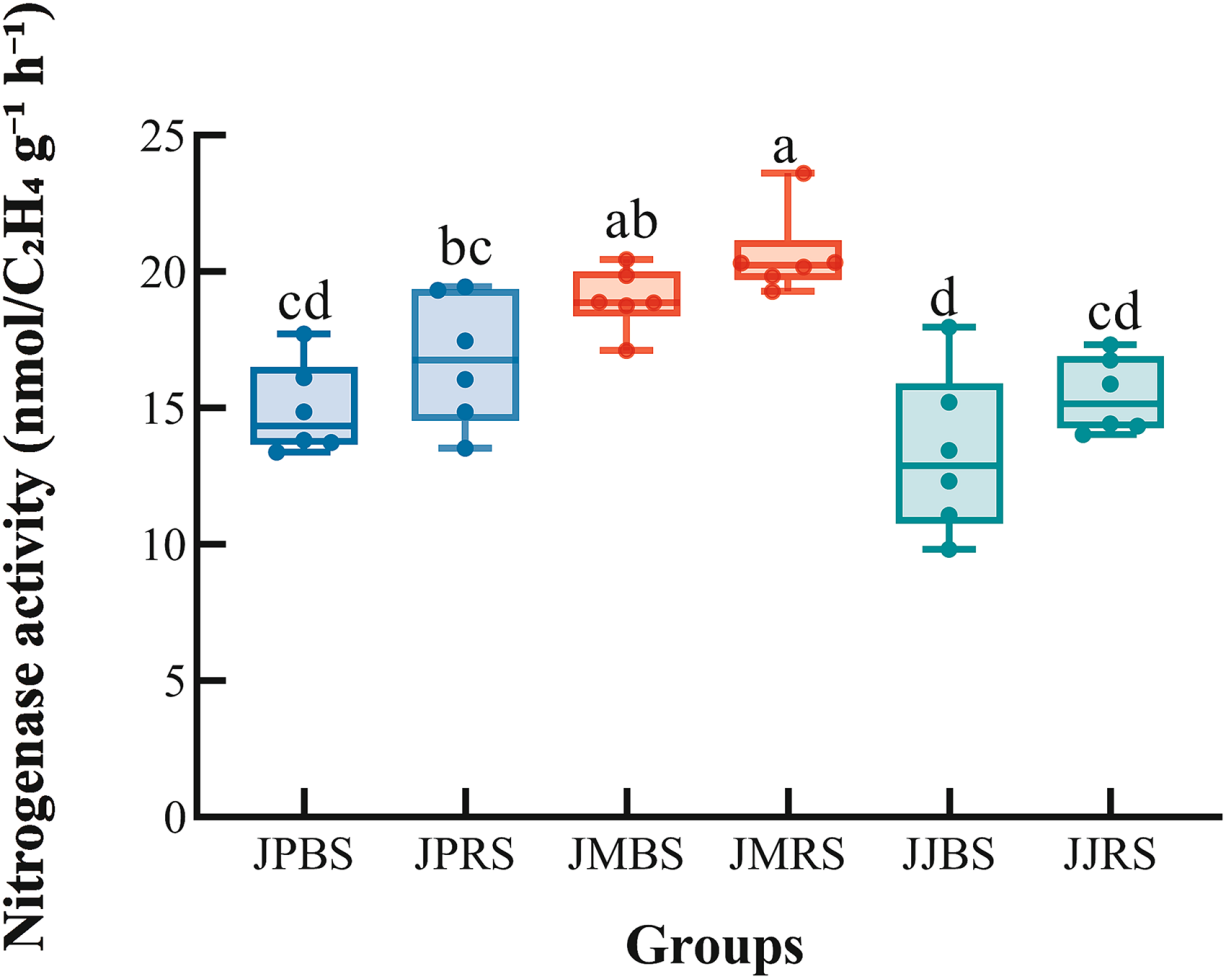
Nitrogenase activity in rhizosphere soil and bulk soil of pecans of different varieties. Boxplot elements: center line = median; box limits = Q3/Q1; whiskers = range. Significant differences (Tukey’s HSD after ANOVA, *p* < 0.05) are indicated by different lowercase letters.

We found that both cultivar and soil compartment significantly affected nitrogenase activity (*p* < 0.01; Table 1). JMBS showed significantly higher nitrogenase activity than JJBS and JPBS (*p* < 0.05), whereas no significant difference was observed between JPBS and JJBS. JMRS exhibited significantly greater activity than JJRS and JPRS (*p* < 0.05), JPRS and JJRS were statistically indistinguishable. Notably, all three cultivars displayed significantly elevated nitrogenase activity in RS compared to BS (Figure 1). In summary, the ‘Mahan’ cultivar showed higher nitrogenase activity in both rhizosphere and bulk soils compared to other cultivars, with consistently greater activity observed in rhizosphere soil versus bulk soil.

**Table 1:** Two-way ANOVA results of nitrogenase activity in rhizosphere soil and bulk soil of pecans of different varieties.

Factors	F	n per group	Partial η^2^	*p*
Varieties	24.592	12	0.621	<0.001
Ecological niche	8.217	18	0.215	0.008
Varieties * Ecological niche	0.059	6	0.004	0.943

The analysis of ecological niches included both rhizosphere and bulk soil compartments, which represent distinct microbial habitats.

### 3.2 Soil *nifH*-harboring community diversity of different pecan cultivars

High-throughput sequencing of the *nifH*-harboring community generated 849,512 high-quality sequences (344,954,731 bases; mean length = 406 bp) from rhizosphere and bulk soil samples across pecan cultivars (Supplementary Table 1). Following rarefaction to 8,413 sequences per sample, sequences clustered into 3,096 OTUs, representing 103 species, 64 genera, 47 families, 32 orders, 16 classes, and 9 phyla. The Scheirer-Ray-Hare test revealed significant differences in Sobs, Shannon, Simpson, and Chao indices among pecan varieties (*p* ≤ 0.05; Table 2), while no significant differences were observed between rhizosphere and bulk soils. Post-hoc comparisons demonstrated that: JM exhibited significantly higher Sobs and Chao indices than JJ, as well as a significantly higher Shannon index than JP (Table 3). Collectively, these α-diversity metrics suggest that pecan varieties exerted a stronger influence on the diversity of soil diazotrophic communities than ecological niche, with the ‘Mahan’ variety consistently demonstrating the highest diversity.

**Table 2:** The Scheirer-Ray-Hare test results of α-diversity indexes of *nifH*-harboring community in rhizosphere soil and bulk soil of pecans of different varieties.

Factors	Sobs			Shannon			Simpson			Chao		
	H	*p*	η^2^	H	*p*	η^2^	H	*p*	η^2^	H	*p*	η^2^
Varieties	6.016	0.05	0.17	6.848	0.03	0.20	6.865	0.03	0.20	6.470	0.04	0.18
Ecological niche	0.361	0.55	0.01	0.121	0.73	0.01	0.121	0.73	0.01	0.361	0.55	0.01
Varieties * Ecological niche	1.146	0.56	0.03	1.307	0.52	0.04	1.545	0.46	0.04	0.697	0.71	0.01

The analysis of ecological niches included both rhizosphere and bulk soil compartments, which represent distinct microbial habitats.

**Table 3:** Multiple comparison results of the soil *nifH*-harboring community indexes in soil of pecans of different varieties.

Indexes	Comparison	ARD	Z	*p*-value	Adjusted *p*
Sobs	JJ vs. JP	−2.500	−0.581	0.561	1.00
	JJ vs. JM	−10.125	−2.354	0.019	0.056
	JP vs. JM	−7.625	−1.773	0.076	0.229
Shannon	JJ vs. JP	7.833	1.821	0.069	0.206
	JJ vs. JM	−3.083	−0.717	0.473	1.000
	JP vs. JM	−10.917	−2.538	0.011	0.033
Simpson	JJ vs. JP	−9.500	−2.209	0.027	0.082
	JJ vs. JM	0.500	0.116	0.907	1.000
	JP vs. JM	10.000	2.325	0.020	0.060
Chao	JJ vs. JP	4.833	−1.124	0.261	0.783
	JJ vs. JM	−10.917	−2.538	0.011	0.033
	JP vs. JM	−6.083	−1.414	0.157	0.472

*Post-hoc* pairwise comparisons were performed following a significant Kruskal–Wallis test. Average rank differences (ARD) is calculated as the mean rank of the first group minus the mean rank of the second group listed in the Comparison column. Standard error (SE) was 4.301 for all comparisons. Z represents the standardized test statistic. Adjusted *p*-values were calculated using the Bonferroni correction for multiple comparisons. All tests are two-sided; significance level *p* = 0.05.

**Figure 2:**
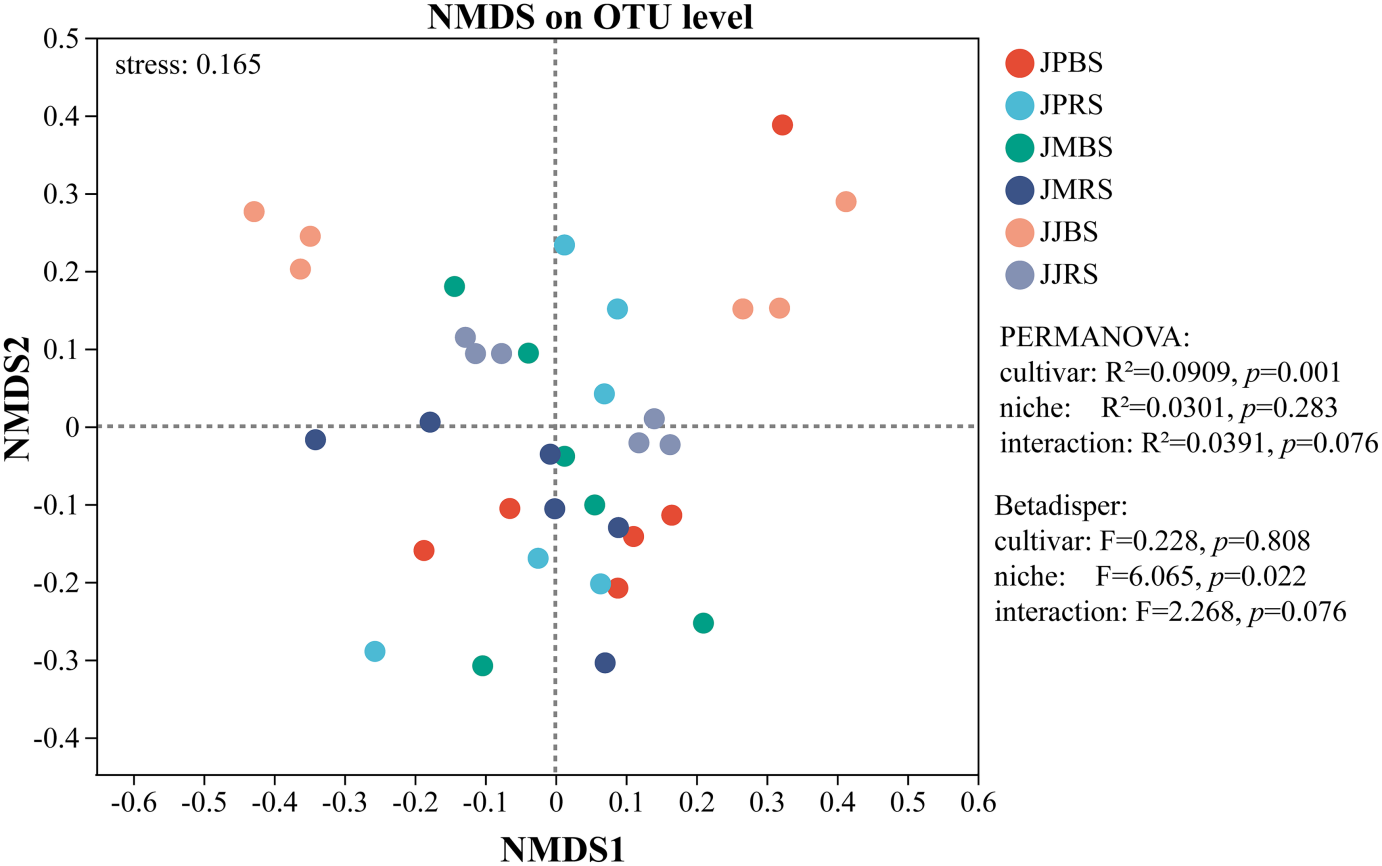
NMDS analysis of the *nifH*-harboring community of in rhizosphere soil and bulk soil of pecans of different varieties. NMDS analysis based on Bray-Curtis distance. Different colors represent distinct sample groups. The closer two sample points are, the more similar their species composition. The *x*-axes and *y*-axes represent relative distances and have no actual units. Stress value: This metric evaluates the reliability of the NMDS ordination. Generally: Stress < 0.2: The NMDS result has reasonable explanatory power. Stress < 0.1: Indicates an excellent ordination with high confidence.

Non-metric multidimensional scaling (NMDS) revealed highly significant differences in *β*-diversity among varieties (*p* < 0.01; Figure 2). Along NMDS2, JJBS samples were distinctly separated from JPBS and JMRS, while NMDS1 showed weaker separation. These results underscore those structural differences in *nifH*-harboring communities were predominantly associated with bulk soil rather than rhizosphere soil across pecan varieties.

### 3.3 Composition and biomarker taxa of the soil *nifH*-harboring community of different pecan cultivars

**Figure 3:**
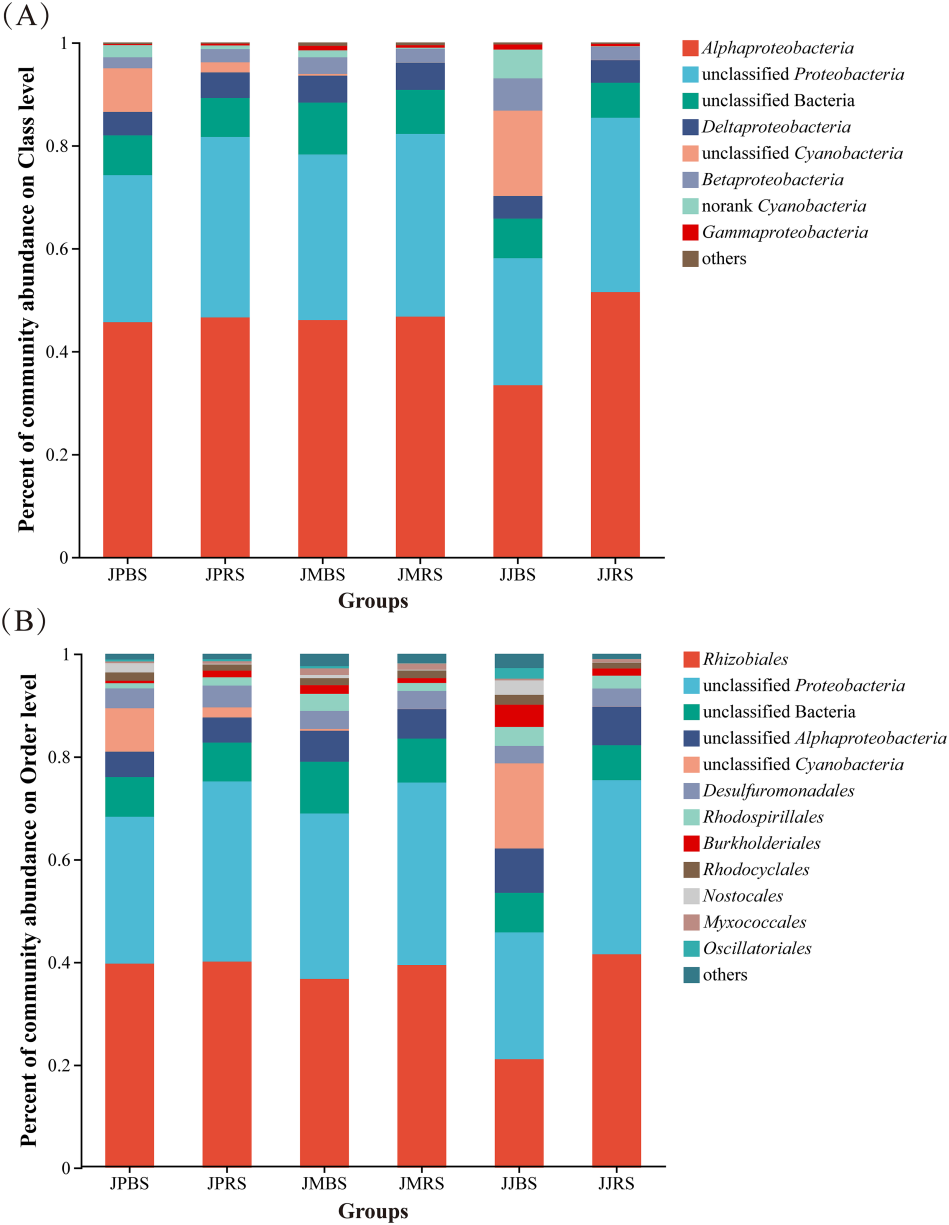
The *nifH*-harboring community compositions of rhizosphere soil and bulk soil of pecans in phylum **(A)** and order **(B)** levels of pecans of different varieties. Taxonomic groups with relative abundances <0.01 were merged and classified as others.

**Figure 4:**
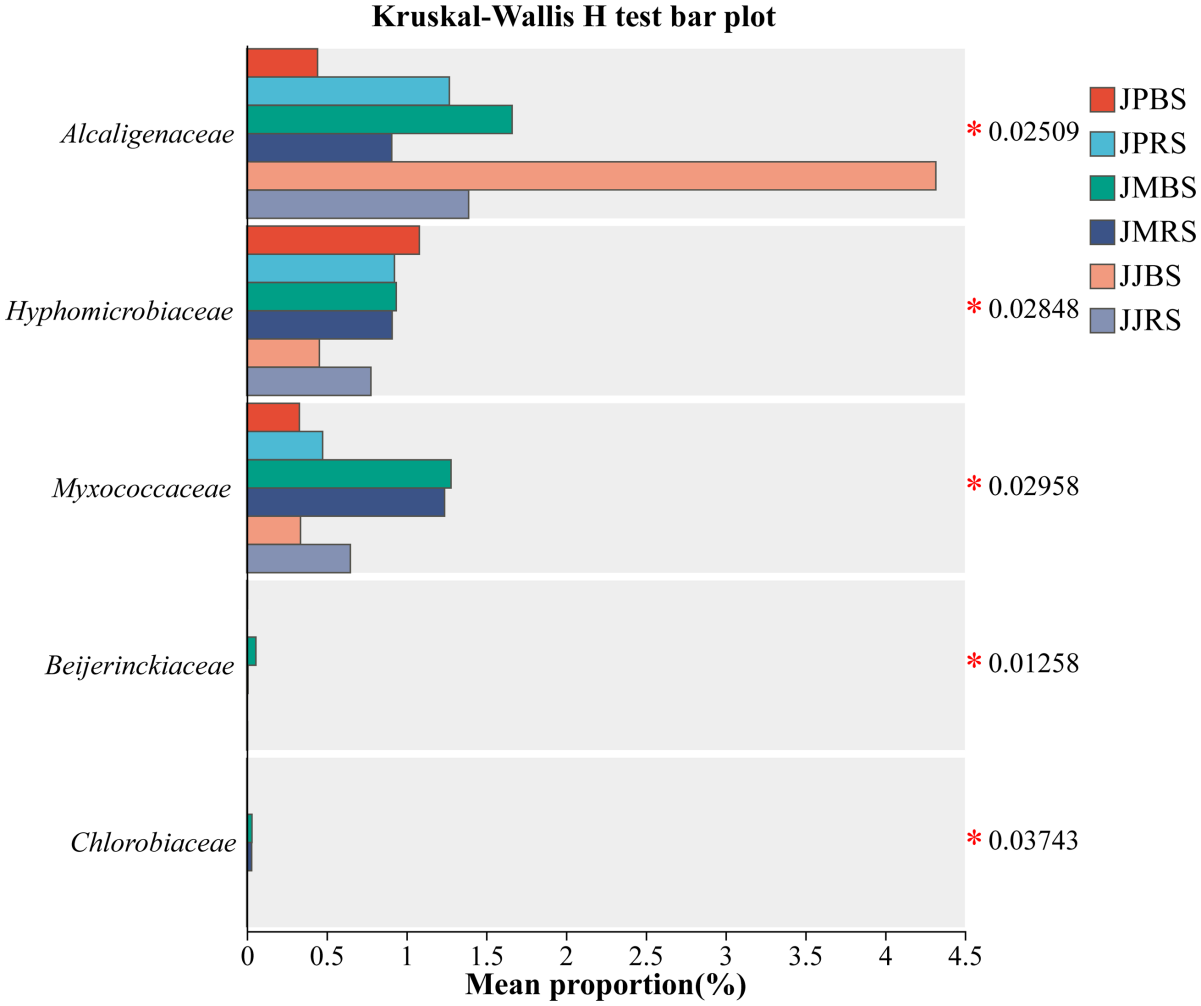
Differences of dominant *nifH*-harboring taxa in rhizosphere soil and bulk soil of pecans of different varieties. Kruskal-Wallis test with FDR correction were used for assessing significance of variety differences. Only the top 10 diazotrophic families in relative abundance are shown (*p < 0.05).

As presented in Figure 3, *Proteobacteria* dominant the *nifH*-harboring community. Notably, JJBS exhibited a distinct microbial profile characterized by significantly lower abundance of *Rhizobiales* and higher representation of *Cyanobacteria* and *Burkholderiales* compared to other groups (Figure 3A). At the order level, *Rhizobiales*, *Desulfuromonadales*, *Rhodospirillales*, *Burkholderiales*, *Rhodocyclales*, *Nostocales*, *Methylococcales*, and *Oscillatoriales* were identified as the dominant bacterial orders, with JJBS demonstrating markedly different community structure from other groups (Figure 3B). Kruskal-Wallis H tests of predominant families revealed significant variations among cultivars. JMBS and JMRS showed significantly higher relative abundances of *Myxococcaceae*, *Beijerinckiaceae*, and unclassified *Betaproteobacteria* compared to other cultivars (*p* < 0.05). JJBS exhibited the highest relative abundance of *Alcaligenaceae* among all groups, while displaying the lowest relative abundances of unclassified *Rhizobiale*s and *Hyphomicrobiaceae* (*p* < 0.05; Figure 4). LEfSe multilevel species discriminant analysis further identified *Rhizobiales* and *Burkholderiales* as biomarker taxa for JJRS and JJBS, respectively (LDA score >4.0, *p* < 0.05; Table 4).

**Table 4:** Biomarkers (at order level) in rhizosphere soil and bulk soil of pecans of different varieties.

Taxa	Groups	LDA	*p*
*Rhizobiales*	JJRS	5.030	0.041
*Burkholderiales*	JJBS	4.297	0.025

Results are LDA-ranked; only orders with *p < 0.05 and LDA > 4.0 shown. Higher LDA = greater species influence.

### 3.4 Network structure of the soil *nifH*-harboring community in different pecan cultivars

**Figure 5:**
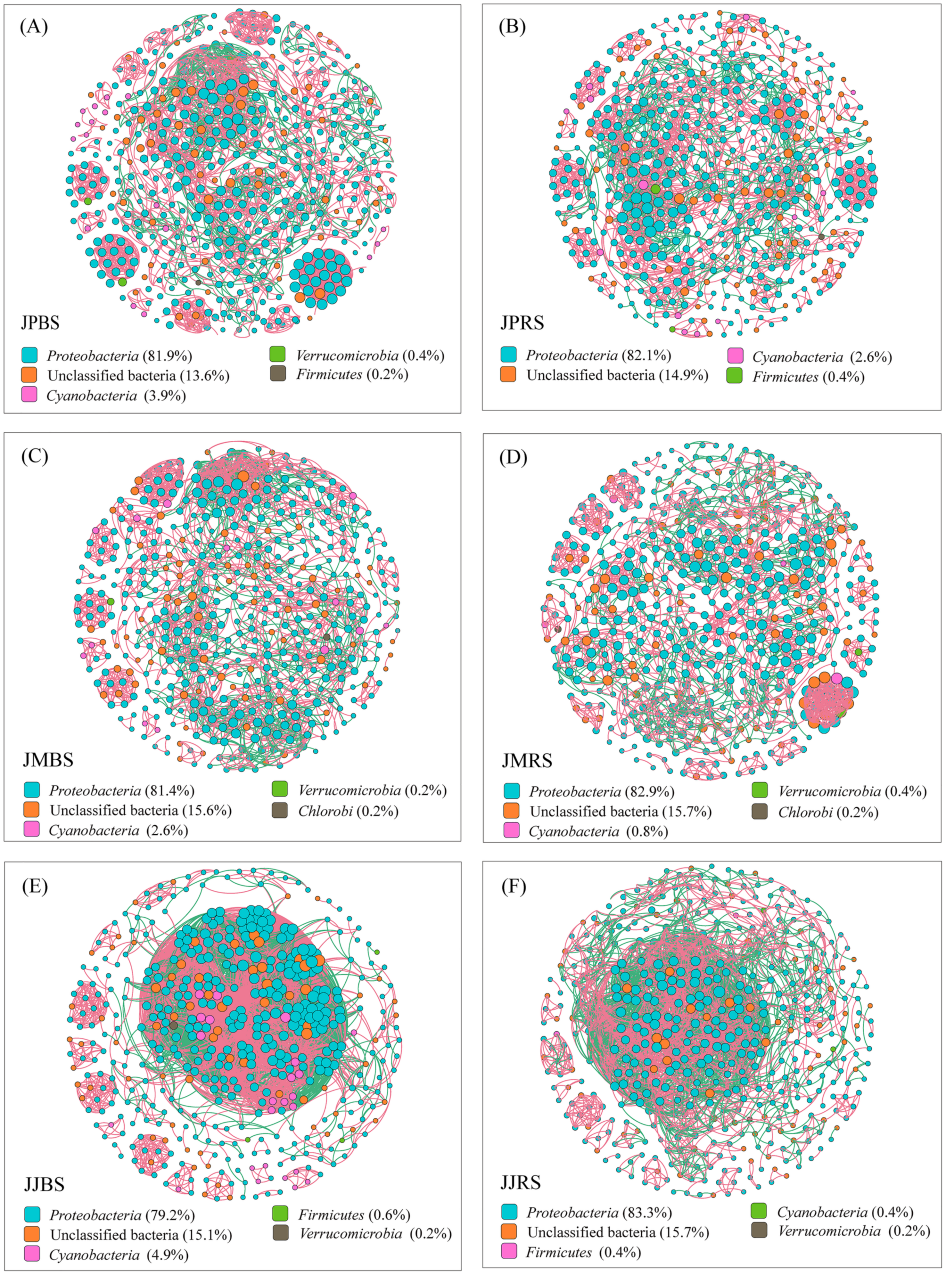
Correlation network analysis of the *nifH*-harboring community in rhizosphere soil and bulk soil of pecans of different varieties. Network of the top 500 OTUs by total abundance. Nodes (OTUs) are colored by phylum and sized by degree. Edges represent strong (Spearman’s *ρ* > 0.8) and significant (*p < 0.01) correlations (red: positive; green: negative). Sample size *n* = 36, covering 3 pecan varieties × 2 ecological niche × 6 replicates.

Network analysis of the top 500 most abundant OTUs revealed distinct topological patterns among pecan cultivars. Both rhizosphere soil and bulk soil of the ‘Jinhua’ cultivar exhibited significantly more complex networks compared to the other two cultivars (Figure 5; Table 5). Specifically, the JJBS network demonstrated the highest total connectivity and average node degree, along with the lowest modularity index, indicating exceptionally tight clustering of bacterial OTUs. These topological characteristics collectively contributed to the increased complexity of the network in JJBS. Comparative analysis showed that rhizosphere soil across all three cultivars consistently formed simpler networks than their bulk soil counterparts, as evidenced by significantly fewer edges, lower node degrees, and reduced overall network complexity (Figure 5; Table 5). Keystone species analysis based on betweenness centrality identified the top five most influential taxa in each group (abundance range: 7–310; Table 6). Taxonomic classification of these keystone taxa revealed distinct patterns across groups: In the JPRS group, the dominant taxa included unclassified *Rhizobiales*, *Azohydromonas*, and *Bradyrhizobium*. The JMBS group was characterized by *Azoarcus* and unclassified *Betaproteobacteria*, while JMRS showed a predominance of unclassified *Rhizobiales*. For the JJBS group, *Rhodomicrobium* and unclassified *Alphaproteobacteria* were prominent, and the JJRS group featured unclassified *Rhizobiales* and *Alphaproteobacteria* as key taxa (Table 6).

**Table 5:** Topological properties of correlation network structure of the *nifH*-harboring community in rhizosphere soil and bulk soil of pecans of different varieties.

Groups	Total nodes	Total links	Positive links	Negative links	Average degree	Modularity	Average clustering coefficient
JPBS	493	2,343	1705	638	9.505	0.808	0.645
JPRS	497	2,220	1,525	675	8.853	0.776	0.631
JMBS	493	2,289	1,516	773	9.286	0.801	0.619
JMRS	496	1998	1,381	615	8.056	0.847	0.618
JJBS	489	7,849	5,546	2,303	32.102	0.434	0.651
JJRS	492	3,691	2011	1,680	15.004	0.529	0.594

**Table 6:** Keystone taxa in correlation network of the *nifH*-harboring community in rhizosphere soil and bulk soil of pecans of different varieties.

Groups	OTU	Abundance	Genus	Phylum	Closeness centrality	Betweenness centrality	Degree
JPBS	OTU3944	68	*unclassified Proteobacteria*	*Proteobacteria*	0.20	4368.58	7
	OTU5435	14	*unclassified Bacteria*	*unclassified Bacteria*	0.21	4221.47	10
	OTU3087	31	*unclassified Proteobacteria*	*Proteobacteria*	0.18	4028.49	4
	OTU3141	117	*unclassified Bacteria*	*unclassified Bacteria*	0.23	3416.05	10
	OTU3555	131	*unclassified Bacteria*	*unclassified Bacteria*	0.23	3328.85	12
JPRS	OTU3944	20	*unclassified Proteobacteria*	*Proteobacteria*	0.24	5234.93	12
	OTU7638	7	*unclassified Rhizobiales*	*Proteobacteria*	0.23	4114.87	14
	OTU3495	47	*Azohydromonas*	*Proteobacteria*	0.22	3947.27	20
	OTU3863	71	*unclassified Bacteria*	*unclassified Bacteria*	0.23	3806.77	19
	OTU5413	201	*Bradyrhizobium*	*Proteobacteria*	0.23	3702.00	17
JMBS	OTU3848	310	*Azoarcus*	*Proteobacteria*	0.21	5955.72	8
	OTU3629	108	*unclassified Proteobacteria*	*Proteobacteria*	0.21	5392.81	9
	OTU902	196	*unclassified Proteobacteria*	*Proteobacteria*	0.21	4708.03	11
	OTU653	12	*unclassified Betaproteobacteria*	*Proteobacteria*	0.21	4093.51	20
	OTU5442	43	*unclassified Proteobacteria*	*Proteobacteria*	0.20	4066.84	10
JMRS	OTU3875	55	*unclassified Rhizobiales*	*Proteobacteria*	0.16	3625.10	6
	OTU525	68	*unclassified Bacteria*	*unclassified Bacteria*	0.20	3345.16	10
	OTU3375	45	*unclassified Bacteria*	*unclassified Bacteria*	0.20	3295.50	12
	OTU2018	14	*unclassified Proteobacteria*	*Proteobacteria*	0.20	3235.53	12
	OTU3226	17	*unclassified Proteobacteria*	*Proteobacteria*	0.18	3229.75	5
JJBS	OTU1729	85	*unclassified Bacteria*	*unclassified Bacteria*	0.26	7881.93	8
	OTU4795	8	*Rhodomicrobium*	*Proteobacteria*	0.21	4274.33	3
	OTU5057	12	*unclassified Bacteria*	*unclassified Bacteria*	0.18	3611.00	5
Groups	OTU	Abundance	*Genus*	*Phylum*	Closeness centrality	Betweenness centrality	Degree
JJBS	OTU1193	52	*unclassified Alphaproteobacteria*	*Proteobacteria*	0.33	3402.02	46
	OTU3829	13	*unclassified Alphaproteobacteria*	*Proteobacteria*	0.26	3261.31	10
JJRS	OTU3979	49	*unclassified Proteobacteria*	*Proteobacteria*	0.25	5634.75	16
	OTU6042	11	*unclassified Alphaproteobacteria*	*Proteobacteria*	0.26	4978.91	15
	OTU3857	57	*unclassified Proteobacteria*	*Proteobacteria*	0.22	4686.11	8
	OTU7868	125	*unclassified Bacteria*	*unclassified Bacteria*	0.20	4532.35	7
	OTU3858	24	*unclassified Rhizobiales*	*Proteobacteria*	0.25	4389.14	11

Higher values of the centrality metrics indicate greater node importance within the network. The table presents the top five species ranked by betweenness centrality, which are identified as keystone taxa in the correlation network. Centrality score ties were handled using the average ranking method.

## 4 Discussion

### 4.1 Cultivar-specific regulation of nitrogen fixation potential and rhizosphere effects

The observed variation in nitrogenase activity among pecan cultivars suggested a potential role of root exudates in modulating the recruitment of free-living diazotroph. This interpretation aligned with established mechanisms in plant-microbe interactions. For instance, plant-secreted flavonoids were shown to enhance the metabolic activity of nitrogen-fixing microorganisms (Yu et al., 2021a; Yan et al., 2022). Similarly, studies in poplar systems demonstrated that host genetic regulation of flavonoid biosynthesis influenced rhizosphere microbiome assembly and promoted nitrogen assimilation through selective microbial enrichment (Wu et al., 2025). It was also recognized that the synthesis of secondary metabolites, including putative signaling molecules, varies according to plant genotype, developmental stage, and physiological status (Bag et al., 2022). Collectively, these findings supporte the hypothesis that cultivar-specific root chemistry mediates microbial nitrogen fixation efficiency by shaping the recruitment of functional microbes. However, direct evidence identifying flavonoids or other exudates as the primary signaling mediators awaits further experimental confirmation.

The energy-intensive nature of biological nitrogen fixation necessitates high-carbon environments (Smercina Darian et al., 2019). Root exudates, rich in low-molecular-weight carbon compounds (sugars, organic acids, and polysaccharides), provide ideal substrates to meet diazotrophic energetic demands (Smercina Darian et al., 2019). Crucially, recent studies suggested carbon substrate quality may be equally important as quantity in regulating fixation rates, with both root exudates and litter inputs potentially driving variation in nitrogen fixation capacity (Wang et al., 2023). In our study, the ‘Mahan’ cultivar demonstrated superior nitrogen fixation potential in both rhizosphere and bulk soils (Figure 1). Analysis of soil chemical properties revealed that the total carbon content in the rhizosphere soil of this cultivar was significantly higher than in other cultivars (Supplementary Table 2). This finding provides a direct mechanistic explanation for the enhanced nitrogen fixation capacity of the ‘Mahan’ cultivar from the perspective of carbon supply, suggesting that it may allocate more carbon resources to the soil through root exudates and litter inputs.

Notably, a consistent rhizosphere effect was observed across all pecan cultivars, as evidenced by significantly higher nitrogen fixation capacity in the rhizosphere than in bulk soils (Figure 1). This functional enhancement was further supported by the elevated total nitrogen (TN) content specifically in the rhizosphere of the ‘Mahan’ and ‘Jinhua’ cultivars (Supplementary Table 2), indicating not only increased nitrogen fixation activity but also actual nitrogen accumulation in the root-associated environment. The rhizosphere effect is likely driven by root-mediated processes such as localized nutrient inputs and the creation of unique physicochemical conditions that facilitate the establishment and function of nitrogen-fixing microorganisms. While previous studies have suggested that factors such as pH, phosphorus availability, micronutrients, and oxygen tension can influence nitrogen fixation (Smercina Darian et al., 2019), the present study provides direct evidence linking rhizosphere properties to both the process and outcome of biological nitrogen fixation in pecan cultivars.

### 4.2 Assembly of *nifH*-harboring communities: diversity, composition, and network complexity

#### 4.2.1 Diversity and community differentiation of *nifH*-harboring communities among cultivars and niches

Our results revealed distinct cultivar-dependent patterns in *nifH*-harboring community assembly. The ‘Mahan’ cultivar demonstrated consistently higher *α*-diversity and nitrogen fixation potential in both rhizosphere and bulk soils (Figure 1; Table 3). We propose two putative mechanisms underlying this observed advantage:

1. Root exudate-mediated microbial recruitment.

Host plants can selectively enrich specific symbiotic diazotroph through root exudates. We speculate that ‘Mahan’ may secrete particular chemical signaling molecules such as flavonoids and benzoic acids, thereby promoting the diazotroph recruitment and activation. The mechanism has been previously documented in maize and sugarcane (Liu et al., 2020; Yu et al., 2021a). Notably, even in bulk soil outside the rhizosphere, ‘Mahan’ maintained higher nitrogen fixation capacity and *nifH*-harboring community diversity (Figure 1; Table 3), suggesting that its root exudates may possess stronger diffusion effects or be capable of inducing a microbial “memory effects” (Haichar et al., 2014; Sasse et al., 2018; Oyserman et al., 2022).

1. Keystone taxa-mediated niche construction contributing to functional stability.

Soils associated with ‘Mahan’ were markedly enriched in keystone taxa from *Myxococcaceae* and *Beijerinckiaceae* (Figure 4), which are known for their dual capabilities in nitrogen fixation and complex carbon metabolism (Borken et al., 2016; Wegner et al., 2019). We propose that this enrichment underpins efficient nitrogen fixation by securing a steady energy supply: the decomposition of complex carbon compounds by these microbes likely provides the necessary substrates and energy (e.g., carbon skeletons and ATP) to support the nitrogenase activity.

#### 4.2.2 Dominant taxa and biomarkers in *nifH*-harboring communities

The predominance of *Proteobacteria* confirms their established role as primary nitrogen-fixing agents in terrestrial ecosystems (Ulrich et al., 2018; Meng et al., 2019; Zhou et al., 2021; Li et al., 2024). However, cultivar-specific partitioning emerged:

Notably, JJBS and JJRS recruited distinct nitrogen-fixing microbiomes, leading to divergent functional outcomes. JJBS exhibited a *nifH*-harboring community enriched with *Cyanobacteria* but depleted in *Rhizobiales* relative to JJRS. This shift toward *cyanobacteria*-mediated pathways may have compromised efficiency, as their photosynthetic oxygen inactivates nitrogenase while their growth intensifies competition for nutrients (Nawaz et al., 2025). Concurrently, JJRS, with its community favoring *α*-*Proteobacteria* (e.g., *Rhizobiales*) for symbiotic fixation, demonstrated superior nitrogenase activity. The key takeaway is that cultivar-specific microbial partitioning is a primary determinant of nitrogen fixation performance. This niche differentiation may be driven by cultivar-specific root exudates or microenvironmental conditions (Hartmann et al., 2009; Yu et al., 2021b; Ulbrich et al., 2022). In this study, the abundance of *Alcaligenaceae* in JJBS was significantly higher (Figure 4), which may reflect specific environmental selection pressure or host physiological state. *Alcaligenaceae*, typically reported as plant growth-promoting bacteria (PGPB; Abdel Latef et al., 2021), are known for heterotrophic nitrogen fixation and stress tolerance, preferring neutral to weakly alkaline environments, with abundance often increasing in high-nitrate conditions (Kalniņš et al., 2020).

In contrast, JMBS and JMRS specifically enriched for *Myxococcaceae* and *Beijerinckiaceae* (Figure 4). The enrichment of these two groups suggests that they may jointly participate in regulating soil nitrogen cycling-potentially through organic matter degradation (*Myxococcaceae*) or free-living nitrogen fixation driven by organic carbon (*Beijerinckiaceae*; Ulrich et al., 2018; Kalniņš et al., 2020; Wolińska et al., 2018; Carrascosa-Robles et al., 2024; Masuda et al., 2024).

LEfSe analysis further confirmed *Rhizobiales* and *Burkholderiales* are the most critical discriminatory biomarker taxa for JJRS and JJBS, respectively (Table 4). In particular, *Burkholderiales* have been recognized as signature *nifH*-harboring community members (Zhou et al., 2021), playing important roles in motility and nitrogen metabolism (Berkelmann et al., 2020). Many associative diazotroph within *Burkholderiales* prefer slightly acidic environments (Dludlu et al., 2018), which is closely related to N content (Zhou et al., 2021). Additionally, many members of the *Burkholderiales* group are renowned for aromatic compound biodegradation potential (Pérez-Pantoja et al., 2012).

Collectively, this evidence points to a clear ecological conclusion: different pecan cultivars adopt distinct nitrogen acquisition strategies by shaping specific rhizosphere microbial communities. JJRS relies on efficient symbiotic nitrogen fixation (*Rhizobiales*), whereas JJBS shifts toward associative or free-living nitrogen fixation pathways driven by *Cyanobacteria* and *Burkholderiales*, likely as an adaptive strategy to low-nitrogen soils. These cultivar-dependent differences in microbial recruitment hold important implications for guiding cultivar selection and formulating precision nutrient management practices. Whether these differences ultimately translate into variations in field-level nitrogen use efficiency warrants further investigation.

#### 4.2.3 Microbial co-occurrence networks and keystone species

Notably, we observed that diazotrophic co-occurrence networks in rhizosphere soils had lower topological features (edge number, node degree) than those in bulk soils (Table 5; Figure 5). This simplification aligns with the established paradigm that strong host plant selection filters rhizosphere communities, reducing complexity and enriching core taxa. Our novel contribution is revealing how this pattern diverges at the cultivar level. Specifically, the exceptionally complex co-occurrence network in JJBS suggests cultivar-specific recruitment of a more interactive microbial consortium (Li et al., 2023), representing a distinct nitrogen acquisition strategy compared to JJRS.

*Proteobacteria* emerged as keystone species in the microbial networks, primarily due to their role in organic substrate mineralization, which supports the nitrogen fixation niche (Xiao et al., 2022). Their network-stabilizing role highlighted the importance of keystone species in regulating cross-kingdom interactions and maintaining nitrogen fixation under environmental fluctuations (Gao et al., 2021). In the JPRS network, *Azohydromonas* and *Bradyrhizobium* occupied central positions (Table 6), indicating a potential direct linke to host plant nitrogen acquisition efficiency. *Bradyrhizobium* is widely recognized for its symbiotic relationship with soybean (Sarao et al., 2025), yet it also functions as a dominant free-living diazotroph (Meng et al., 2019; Zhou et al., 2021; Li et al., 2022). Similarly, *Azohydromonas* was commonly detected prevalence in *nifH*-harboring community (Xie et al., 2021; Tang et al., 2023).

We identified *Azoarcus* and Rhodomicrobium as network hubs in JMBS and JJBS, respectively, (Table 6). *Azoarcus* is a well-characterized free-living diazotroph (Sarao et al., 2025), can form associative nitrogen-fixing relationships with plants to promote growth (Sakoda et al., 2019), and has been found enriched in rice endophytic communities (Wartiainen et al., 2008). The prominence of these non-symbiotic diazotrophs in ecological networks may reflect the ecological relevance of chemoheterotrophic nitrogen fixation pathways in bulk soils. Furthermore, the frequent detection of unclassified *Rhizobiales* as core species indicates certain limitations in current taxonomic databases for the functional interpretation of environmental microbes. For instance, this may lead to provisional functional annotations or obscure the specific ecological functions of certain uncultivated taxa. These observations further underscore the need to integrate multi-omics approaches in future research to more comprehensively elucidate the ecological roles and functional potential of these uncultivated microbial lineages.

### 4.3 Implications for pecan cultivation and future research directions

These findings provide novel insights for cultivar selection from the perspective of microbiome-mediated nitrogen acquisition optimization. Subsequent studies will integrate metabolomics with gnotobiotic cultivation experiments to establish causal relationships between root exudates and diazotroph recruitment. Our study demonstrated that cultivar selection modulated nitrogen fixation potential through microbial community restructuring. For pecan cultivation, promoting genotypes with strong rhizosphere effects (e.g., ‘Mahan’) may enhance soil nitrogen availability while reducing dependence on synthetic fertilizers.

However, the functional significance of taxonomic biomarkers requires further validation through metagenomics and stable isotope probing techniques to link community structure with *in situ* nitrogen fixation rates. Future investigations should also examine: (1) long-term interactions between cultivar-driven microbial communities and soil physicochemical properties, and (2) the feasibility of inoculating superior diazotroph to enhance nitrogen fixation capacity in low-performance cultivars.

In conclusion, this study revealed cultivar-specific strategies for recruiting and maintaining diazotrophic communities in pecan systems, with significant implications for precision agriculture and microbial resource management. The integration of plant genotype selection with soil microbiome engineering may offer a promising pathway for optimizing nutrient cycling and advancing sustainable pecan production.

## 5 Conclusion

This study demonstrates that pecan cultivars play a decisive role in shaping the structure of rhizosphere diazotrophic communities. ‘Mahan’ cultivar specifically enriched key nitrogen-fixing taxa, including *Bradyrhizobium* and *Azoarcus*, and reinforced the interaction stability of the microbial network. These findings provide a mechanistic, microbe-mediated explanation for the enhanced nitrogen-cycling potential associated with superior cultivars. Our results suggest that selecting cultivars with a high capacity for recruiting beneficial diazotrophs could serve as an ecologically informed strategy to reduce dependence on synthetic nitrogen fertilizers. Future work should prioritize elucidating the molecular drivers, such as cultivar-specific root exudate profiles, that govern microbial assembly, and conducting long-term field trials to validate whether the identified microbial biomarkers can reliably predict in-situ nitrogen fixation capacity. Such efforts will provide a robust scientific basis for microbiome-based management in pecan cultivation.

## Data Availability

The datasets presented in this study can be found in online repositories. The names of the repository/repositories and accession number(s) can be found in the article/Supplementary material.

## Footnotes

1
http://fungene.cme.msu.edu/

2
https://cloud.majorbio.com/
