# Supplementary material for: Soil nifH-harboring community assemblage varies across pecan cultivars
Source: Front Microbiol. 2026 Jan 7;16:1716240. doi: 10.3389/fmicb.2025.1716240 (PMC12819599; doi:10.3389/fmicb.2025.1716240)
Supplement: Supplementary file 1 [file Table_1.docx]

Supplementary Table 1 Sequencing information statistics in different groups

| Sample ID | Group | Effective tags | Total bases | Average sequence length |
| --- | --- | --- | --- | --- |
| 1 | JPBS | 24070 | 9778081 | 406 |
| 2 |  | 24988 | 10153353 | 406 |
| 3 |  | 24843 | 10083118 | 406 |
| 4 |  | 24535 | 9966314 | 406 |
| 5 |  | 24240 | 9839866 | 406 |
| 6 |  | 23194 | 9429524 | 407 |
| 31 | JPRS | 24694 | 10044539 | 407 |
| 32 |  | 17539 | 7122184 | 406 |
| 33 |  | 19668 | 7977318 | 406 |
| 34 |  | 23454 | 9521481 | 406 |
| 35 |  | 19759 | 8038392 | 407 |
| 36 |  | 24078 | 9795999 | 407 |
| 7 | JMBS | 24985 | 10142139 | 406 |
| 8 |  | 23249 | 9441401 | 406 |
| 9 |  | 23667 | 9610980 | 406 |
| 10 |  | 23596 | 9585282 | 406 |
| 11 |  | 23627 | 9596469 | 406 |
| 12 |  | 24539 | 9959239 | 406 |
| 37 | JMRS | 24231 | 9827666 | 406 |
| 38 |  | 24400 | 9894049 | 405 |
| 39 |  | 23643 | 9613761 | 407 |
| 40 |  | 24898 | 10110832 | 406 |
| 41 |  | 23220 | 9426830 | 406 |
| 42 |  | 23611 | 9589397 | 406 |
| 13 | JJBS | 24671 | 10018078 | 406 |
| 14 |  | 23564 | 9560288 | 406 |
| 15 |  | 24718 | 10032926 | 406 |
| 16 |  | 23555 | 9564037 | 406 |
| 17 |  | 23239 | 9437471 | 406 |
| 18 |  | 23793 | 9657103 | 406 |
| 43 | JJRS | 23501 | 9542841 | 406 |
| 44 |  | 23965 | 9729963 | 406 |
| 45 |  | 23017 | 9342490 | 406 |
| 46 |  | 23584 | 9571936 | 406 |
| 47 |  | 24212 | 9826858 | 406 |
| 48 |  | 24965 | 10122526 | 405 |
| 合计 |  | 849512 | 344954731 | 406 |

Sample IDs correspond to the original data accession numbers in NCBI.

Supplementary Table 2 Multiple comparison results of the soil chemical properties in different groups

| Groups | pH | TC  g/kg | TN  g/kg | NH_4_^+^-N mg/kg | NO_3_--N mg/kg | AP  mg/kg | AK  mg/kg | TP  g/kg | TK  g/kg |
| --- | --- | --- | --- | --- | --- | --- | --- | --- | --- |
| JPBS | 6.22±0.38a | 10.37±1.46b | 1.42±0.57  ab | 7.20±2.6  c | 3.36±0.8  a | 32.81±11.2  a | 171.39±47.4  a | 1.19±0.1  ab | 11.76±3.7  a |
| JPRS | 6.49±0.64a | 11.13±1.75  ab | 1.15±0.62  ab | 9.99±1.1  ab | 3.70±0.7  a | 43.73±8.28  a | 185.08±68.4  a | 1.29±0.1  a | 10.02±0.6  a |
| JMBS | 6.25±0.44a | 12.35±1.49  ab | 0.93±0.29  ab | 10.5±2.1  ab | 2.81±1.4  a | 32.95±8.75  a | 191.12±18.6  a | 1.17±0.1  ab | 12.88±4.9  a |
| JMRS | 6.30±0.72a | 13.73±1.37a | 1.60±0.40  a | 12.2±0.7  a | 2.40±0.6  a | 37.44±8.65  a | 173.11±12.5  a | 1.22±0.1  ab | 15.98±4.4  a |
| JJBS | 6.14±0.64a | 11.08±2.05  ab | 0.65±0.61  b | 7.22±0.4  c | 3.19±1.1  a | 40.80±18.0  a | 190.79±56.7  a | 1.15±0.1  b | 10.82±0.1  a |
| JJRS | 6.22±0.28a | 11.47±1.12  ab | 1.40±0.28  ab | 8.03±0.6  bc | 2.83±1.0  a | 41.11±17.5  a | 220.02±74.6  a | 1.16±0.1  ab | 14.20±2.9  a |

Data are presented as mean ± standard deviation (Mean ± SD). Different lowercase letters within the same column indicate significant differences among groups based on Tukey's test (p < 0.05).

| Soil properties | RDA1 | RDA2 | r2 | p_values |
| --- | --- | --- | --- | --- |
| TC | 0.2448 | -0.9696 | 0.0598 | 0.345 |
| TN | -0.9834 | -0.1812 | 0.1036 | 0.174 |
| TP | -0.0483 | 0.9988 | 0.1503 | 0.063 |
| TK | 0.603 | -0.7977 | 0.0226 | 0.703 |
| NH4+-N | -0.9778 | -0.2097 | 0.0679 | 0.313 |
| NO3－-N | 0.9065 | 0.4221 | 0.1015 | 0.178 |
| AP | 0.9943 | 0.1068 | 0.0584 | 0.371 |
| AK | 0.4395 | -0.8982 | 0.1323 | 0.093 |
| pH | -0.9541 | 0.2996 | 0.007 | 0.891 |

Supplementary Table 3 esults of the envfit analysis for environmental variables

An envfit analysis (999 permutations) to test the significance of the correlations between environmental variables and microbial community structure.





Supplementary Figure 1 Sequencing information statistics in different groups


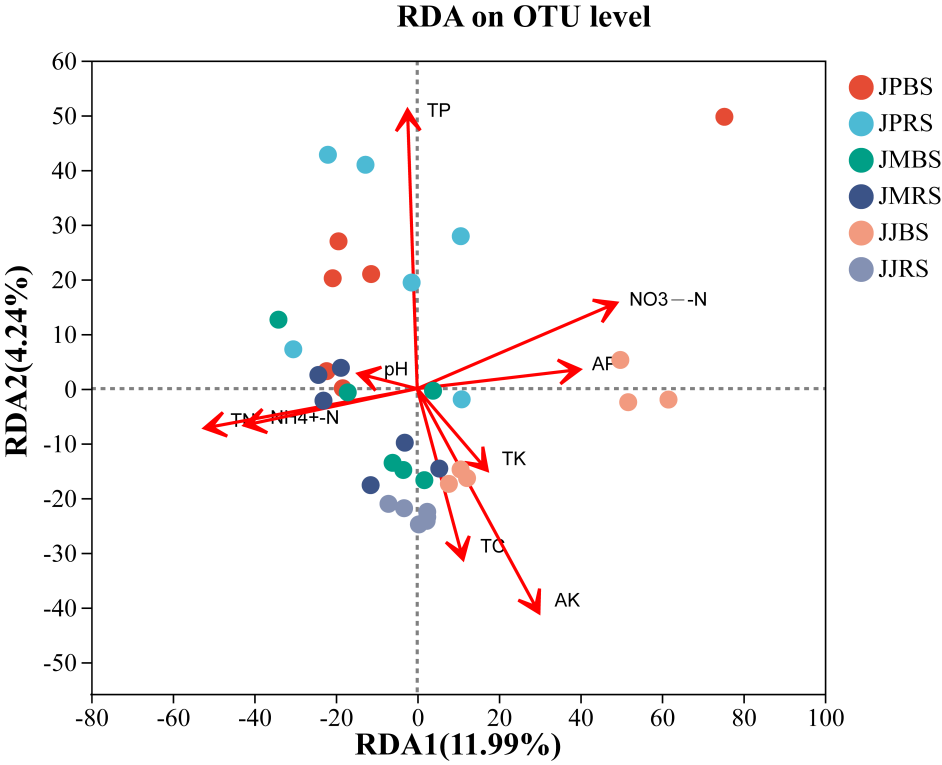


Supplementary Figure 2 RDA analysis between *nifH-*harboring community structure and soil properties.

Points represent samples (colored/shaped by environment); arrows indicate environmental factors. Arrow length corresponds to factor-community correlation; angle with axis reflects factor-axis correlation.
